# Aging impact on canine extracellular vesicles production, size, and miRNA content

**DOI:** 10.1007/s11259-026-11354-6

**Published:** 2026-06-19

**Authors:** Clara Luna Marina, Alexandra Mazer Greuel, Lucas de O. Las-Casas, Evilly Lopes-Gomes, Sabrina Simplício de Araújo Romero Ferrari, Marcio Lourenço Rodrigues, Flavia C. G. dos Reis, Anamélia Lorenzetti Bocca, Simoneide Souza Titze-de-Almeida, Fernando Francisco Borges Resende, Fabiano José Ferreira de Sant’Ana, Ricardo Titze-de-Almeida

**Affiliations:** 1 Technology for Gene Therapy Lab., University of Brasília, Brasília, Federal District Brazil; 2 Carlos Chagas Institute, Oswaldo Cruz Foundation, Curitiba, Paraná Brazil; 3 Instituto de Microbiologia Paulo de Goes, Federal University of Rio de Janeiro, Rio de Janeiro, Brazil; 4 Centre for Medical Mycology in Latin America (CMM LATAM) Unit, São Paulo, Brazil; 5 Bi-Institutional Platform for Translational Medicine, Oswaldo Cruz Foundation, Ribeirão Preto, São Paulo, Brazil; 6 National Institute of Science and Technology in Human Pathogenic Fungi, Ribeirão Preto, São Paulo, Brazil; 7 Molecular Biotechnology Center, University of Brasilia, Brasília, Federal District Brazil; 8 University Center of the Central Highlands Apparecido dos Santos (UNICEPLAC), Gama, Brasília, Federal District Brazil

**Keywords:** Extracellular vesicles, Aging, Plasma, Dog, miRNA

## Abstract

Increasing life expectancy has been accompanied by a higher prevalence of age-associated conditions, underscoring the need to better understand biological mechanisms of aging and to identify accessible biomarkers. Extracellular vesicles (EVs) are lipid-bilayer vesicles that mediate intercellular communication by transporting proteins, lipids, and regulatory RNAs, including microRNAs (miRNAs). Here, we investigated whether the abundance, morphology, and molecular cargo of plasma-derived EVs differ between young and senior dogs. Blood samples were obtained from 24 clinically healthy dogs in Gama (Federal District, Brazil), and plasma EVs were isolated by sequential centrifugation, filtration through a 0.22 μm filter, and ultracentrifugation. EVs concentration and size distribution were assessed by nanoparticle tracking analysis (NTA), morphology by transmission electron microscopy (TEM), and cargo by quantification of total protein and sterols using commercial assays. In addition, we quantified miR-19b, miR-29c, miR-7, miR-155, and miR-21 by Real Time Quantitative Polymerase Chain Reaction (RT-qPCR). Senior dogs exhibited a lower plasma EV yield and greater size heterogeneity, with a higher proportion of larger EVs. Total protein and sterol content per starting plasma volume were reduced in the senior group; however, sterol normalized per EV was increased, consistent with compositional remodeling of circulating vesicles with age. Finally, EV-associated miRNA levels were reduced in senior dogs, particularly miR-19b and miR-29c. Collectively, these findings indicate that canine aging is associated with marked changes in plasma EV abundance, morphology, and cargo, indicating that EVs could represent a promising tool for investigating age-related disorders in dogs.

## Introduction

As life expectancy increases, population aging has become an increasingly important concern, accompanied by a growing elderly population and a consequent rise in the prevalence of neurodegenerative disorders and other associated diseases and disabilities (Martinez et al. 2021, Guo et al. 2022, López-Otín et al. 2023). As a result, older adults may experience a decline in quality of life that also affects their families, while increasing strain on healthcare systems and exerting broader economic impacts (Acemoglu and Johnson 2007). A similar pattern is observed in veterinary medicine, where increasing pet life expectancy has been accompanied by a broad spectrum of age-associated conditions, including ocular, cardiac, orthopedic, neoplastic, and neurodegenerative disorders (Montoya et al. 2023, Fefer et al. 2022, Nam et al. 2024).

The biological basis of aging is complex; however, the process can be understood as the cumulative consequence of natural failures in the mechanisms that maintain homeostasis (López-Otín et al. 2023, Bellows et al. 2015). These failures could be studied in humans and animals to better understand the process and identify targets for new therapies that may consequently increase the quality of life of elderly individuals and animals (Wang et al. 2023). An important hallmark is genomic instability, characterized by DNA replication errors, chromosome segregation defects, oxidative processes and spontaneous hydrolytic reactions that cannot be corrected by the DNA repair machinery, leading to the accumulation of significant mutations (Yang et al. 2024, Vijg and Dong 2020). Other aging hallmark is the progressive telomeres shortening, a consequence of the incapacity of DNA polymerases to complete the copy of telomeric regions of eukaryotic DNA during replication, which can lead to apoptosis or cellular senescence when telomeres become too short (Blackburn et al. 2015). Normally, this process is delayed by telomerase and failure in this enzyme leads to accelerated aging and neurodegenerative disorders such as Alzheimer’s disease (Shim et al. 2021, Hipp et al. 2019, Pievani et al. 2014, Martínez-CuNoemí Rueda et al. 2020, Zhang et al. 2025).

Given the role of pathological proteins in aging processes, it is essential to examine the cellular mechanisms underlying their transport, which can be carried out by exosomes. Exosomes are small extracellular vesicles (EVs) formed by a membranous lipid bilayer (30 to 100 nm) and produced within the endosomal system (Titze-de-Almeida et al. 2025). EVs are secreted by almost all cells and are responsible for cell-to-cell communication (Théry et al. 2018). EVs carry a plenty of proteins, enzymes and ncRNAs that are released into the cytosol of target cells, regulating gene expression and post-transcriptional modifications, which directly influence important biological processes (Hamdan et al. 2021). Recently, exosomes have emerged as promising candidates for the treatment of tissue dysregulation or as disease biomarkers, as they are abundant in biological fluids and provide information about the organ or cell from which they were produced, through their surface or content molecules (Théry et al. 2018, Titze-de-Almeida et al. 2025).

The role of EVs in aging has been increasingly explored (Manni et al. 2023, Safaei et al. 2025). Studies in humans show that senescent and damaged cells produce more EVs than younger ones; however, controversially, the plasma EVs concentration decline with age (Hamdan et al. 2021). Furthermore, treating senescent cells with EVs derived from pluripotent stem cells reduced the reactive oxygen species production and ameliorated the skin aging phenotype (Oh et al. 2018). Nonetheless, the characteristics of EVs during canine aging have not been previously described, and dogs represent a reliable translational model for age-related disorders in humans (Ruple et al. 2025, Panek et al. 2020).

The present study aimed to analyze extracellular vesicles (EVs) isolated from the plasma of young and senior dogs, in order to investigate age-related differences in their characteristics and molecular cargo. EVs, including exosomes, microvesicles, and liposomes, play a crucial role in maintaining physiological processes, as they are essential for intercellular communication. These vesicles transport a variety of molecules involved in signaling, feedback regulation, and signal transduction, thereby contributing to the maintenance of organismal homeostasis (Théry et al. 2018, Hamdan et al. 2021, Titze-de-Almeida et al. 2025, Gennebäck et al. 2013). Based on this, circulating EVs may serve as valuable sources of information regarding the physiological state of an organism and may even provide insights into organ-specific dysfunction. Furthermore, we hypothesize that both the characteristics and molecular content of EVs change significantly with aging, highlighting their potential as biomarkers for age-related diseases. To address this, plasma samples were collected from 24 dogs in the Federal District, Brazil, which were stratified into young and senior groups. EV-enriched fractions were then isolated to compare EV yield, morphological features, and miRNA content between the two cohorts. We selected miRNAs related to aging process and associated diseases.

## Methodology

### Sampling population

Samples were collected from clinically healthy dogs in Gama, an administrative region of the Federal District (DF), Brazil, through the Veterinary Clinical Neurology Service of the Centro Universitário do Planalto Central Apparecido dos Santos (UNICEPLAC). Owners were invited to authorize the donation of a blood sample for inclusion in the study. The protocol was approved by the Ethics Committee on Animal Use of the University of Brasília (CEUA/UnB: 23106.109701/2024-4) and by the UNICEPLAC Ethics Committee (CEUA/UNICEPLAC: 012/2024). The study population was assembled as two age-defined cohorts of clinically healthy, small-sized dogs (< 10 kg) to minimize potential allometric confounding related to body size. Age cutoffs were chosen based on established canine life-stage frameworks and to maximize contrast between age extremes in this exploratory design (Bartges et al. 2012). The young/control group comprised 12 dogs aged 2–6 years, including predominantly mixed-breed animals (*n* = 10) and two purebred dogs (one Shih-tzu and one Spitz). The senior group comprised 12 small-sized dogs aged with more than 12 years, also with body weight < 10 kg, including eight mixed-breed dogs and four purebred dogs (one Shih-tzu, one Shipperke, one Bichon Frise and one Chihuahua), as described on Table 1. Overall, both cohorts were therefore dominated by mixed-breed animals, a demographic pattern typical of the local canine population, while maintaining comparable small-body-size composition across groups; we acknowledge that, although this reduces the likelihood that results are driven by a single breed-specific EV phenotype, residual variability related to mixed genetic backgrounds and the absence of an intermediate adult group may contribute to dispersion and should be addressed in future breed- and age-stratified studies (Nam et al. 2024, Bartges et al. 2012, Dias et al. 2016, Mendonça et al. 2025).

**Table 1:** Dogs enrolled in the study and sample destination for each analysis

Animal ID	Sex	Breed	Age	Microscopy/NTA	Protein / Sterol content	RT-qPCR
1	Female	Mixed	4	X	X	X
2	Female	Mixed	6	X		X
3	Female	Shih-tzu	2		X	X
4	Female	Mixed	4		X	X
5	Female	Mixed	3			X
6	Female	Mixed	3			X
7	Male	Mixed	2	X	X	X
8	Male	Mixed	2	X		X
9	Male	Mixed	3		X	X
10	Male	Spitz	3		X	X
11	Male	Mixed	4	X		X
12	Male	Mixed	4	X		X
13	Female	Bichon frise	12			X
14	Female	Mixed	15		X	X
15	Female	Mixed	12	X	X	X
16	Female	Chihuahua	15		X	X
17	Female	Mixed	> 12	X	X	X
18	Female	Mixed	> 12	X		X
19	Female	Shipperke	14			X
20	Female	Mixed	> 12	X		X
21	Male	Mixed	> 12	X		X
22	Male	Shih-tzu	15			X
23	Male	Mixed	> 12	X	X	X
24	Male	Mixed	13		X	X

### EVs isolation

For EVs isolation, we collected at least 5 mL of blood from dogs using EDTA-containing tubes. The blood samples were centrifuged at 300 g for 5 min, and the plasma was collected from the upper liquid phase. Next, 500 µL of plasma was then centrifuged at 10,000 g for 10 min to eliminate debris and residual cells, and the supernatant was filtered through 0.22 μm filters and ultracentrifuged at 100,000 g for 1.5 h. The supernatant was discarded, and the EV-enriched pellet, resuspended in Phosphate-Buffered Saline (PBS). The ultracentrifugation was repeated twice, and the final pellet containing exosomes was resuspended in 150 µL PBS.

### EVs characterization by yield, size and content

We characterized the EVs by assessing their protein content using the Micro BCA Protein Assay Kit (Thermo Fisher) and sterol content with the Amplex Red Cholesterol Assay Kit (Thermo Fisher), following the manufacturers’ recommendations. We measured yield and size through Nanoparticle Tracking Analysis (NTA), and we examined their morphology and size using Transmission Electron Microscopy (TEM), as detailed below.

### EVs’ quantification by nanoparticle tracking analysis (NTA)

The quantification of EVs was done by NTA (LM10) system coupled to a 488-nm laser, equipped with a camera and flow pump (Malvern Panalytical, Malvern, United Kingdom) and the NTA 3.0 software (Malvern Panalytical). The samples were injected with 1-mL syringes attached to a continuous flow injection pump. Three 60-second videos (camera level at 15, gain at 3) were obtained per sample after the passage of the samples through the light beam. The viscosity of the samples was maintained as that of water. For data analysis, the camera gain was changed to 10 − 15, and the detection limit used was three for all samples. If necessary, the samples were diluted in PBS to achieve the optimal range of 9 × 10^7^ to 2.9 × 10^9^ particles/mL (Peres da Silva et al. 2018).

### EVs’ morphological analysis by transmission electron microscopy (TEM)

For optical analysis of the EVs, the isolated samples were observed using TEM. After homogenization, 50 µL of the EVs suspensions were added to Formvar-coated grids to allow adherence for 60 min at room temperature. Then, the grids were washed with 30 µL of sterile PBS, and the excess buffer was dried with filter paper. The grids were then incubated with 30 µL of Karnovski solution for 10 min, washed three times with cacodylate buffer, and finally dried with filter paper. The samples were counterstained with 5% uranyl acetate for 2 min. The grids were washed once with H_2_O, dried with filter paper, and transferred to a metallizer (Leica EM ACE200), where they were covered with carbon particles for later visualization with a JEOL 1400 Plus microscope with beam acceleration at 90 kV (Castelli et al. 2022). Images were analyzed and particles were measured for diameter, membrane thickness and electron density using the software Image J.

### miRNA quantification

For miRNA quantification, we first extracted the miRNA fraction from the EVs samples using the miRNeasy Mini Kit (Qiagen), following the manufacturer’s recommendations. The resulting miRNA isolates were quantified using the Qubit microRNA Assay Kit (Invitrogen), and the volumes were adjusted to ensure the same amount of miRNA for all samples. Then, they were reverse transcribed to cDNA using the TaqMan Advanced miRNA cDNA synthesis kit (Thermo Fisher), in accordance with manufacturer’s instructions, and quantified by real time polymerase chain reaction (RT-qPCR) Taqman system (QuantStudio 12 K Flex system, Thermo Fisher). The reaction mix contained 2 µl cDNA, 1 µl miRNA specific FAM dye-labeled TaqMan primers [miR-19b (478264_mir), miR-29c (479229_mir), miR-7 (483061_mir), miR-155 (483064_mir), miR-21 (477975_mir) and as endogenous controls we used the primers miR-39 (478293_mir) and miR-320a (478594_mir), all from Thermo Fisher Scientific], 10 µl TaqMan Fast Advanced Master Mix (Thermo Fisher Scientific) and milli-Q pure water to 20 µl. The qPCR program consisted of two initial cycles (50 °C for 2 min, 95 °C for 20 s), followed by 40 amplification cycles (95 °C for 1 min, 60 °C for 1 min). Each reaction was run in triplicate, including water as negative control. Relative expression of microRNAs was calculated by the delta–delta Ct method (2^−ΔΔCt^) (Horst et al. 2018, Livak and Schimittgen et al. 2001).

### Statistical analysis

The figures in the present study are representative of at least three technical and biological replicates, comprising three independent experiments. The data are presented as the average of the obtained values and their standard error of the mean (SEM). We analyzed if the data followed a normal distribution using Shapiro–Wilk test and statistical analysis was conducted using unpaired T-test, a statistical test to compares two groups in the software GraphPad Prism, version 9. Significant statistical differences were considered when *P* < 0,05. *P*-values represented by *P < 0.05, **P < 0.01, ***P < 0.001, and ****P < 0.0001.

## Results

### Senior dogs produce fewer EVs than young dogs

**Fig. 1:**
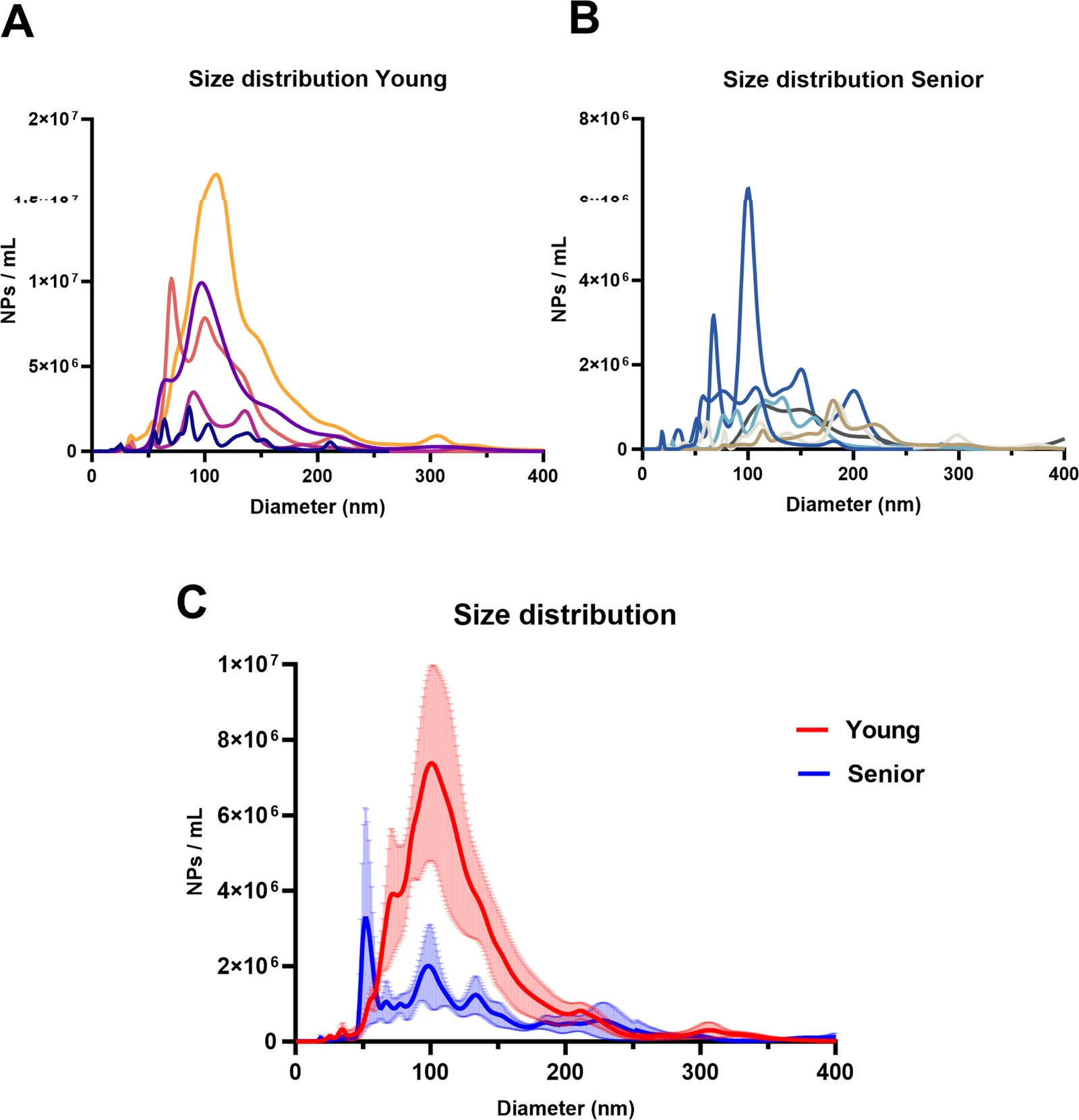
Senior dogs produce less and more diverse EVs than young dogs. Size distribution and concentration of nanoparticles (Np) measured by Nanoparticle Tracking Analysis (NTA) comparing plasma EVs from dogs aged 2 to 6 years (young) (**A**) and dogs over 12 years (senior) (**B**). In panels a and b, each curve color represents an individual dog donor, showing the prevalence of Np within each size range. In panel **C**, the line represents the mean of Np size distribution comparing EVs from young and senior dogs, while the shaded area indicates the standard error of the mean (SEM)

After collecting plasma from young and senior dogs and isolating their EVs, we analyzed nanoparticles concentration and size distribution using NTA. In Fig. 1A and B, we noted great diversity in nanoparticles’ size and concentration among individuals. However, when considering all samples together, as shown in Fig. 1C, we observed that EVs from young dogs are homogeneous in size, forming a major peak around 100 nm in diameter, whereas EVs from elderly dogs are more diverse, with peaks ranging from 50 nm to 250 nm.

**Fig. 2:**
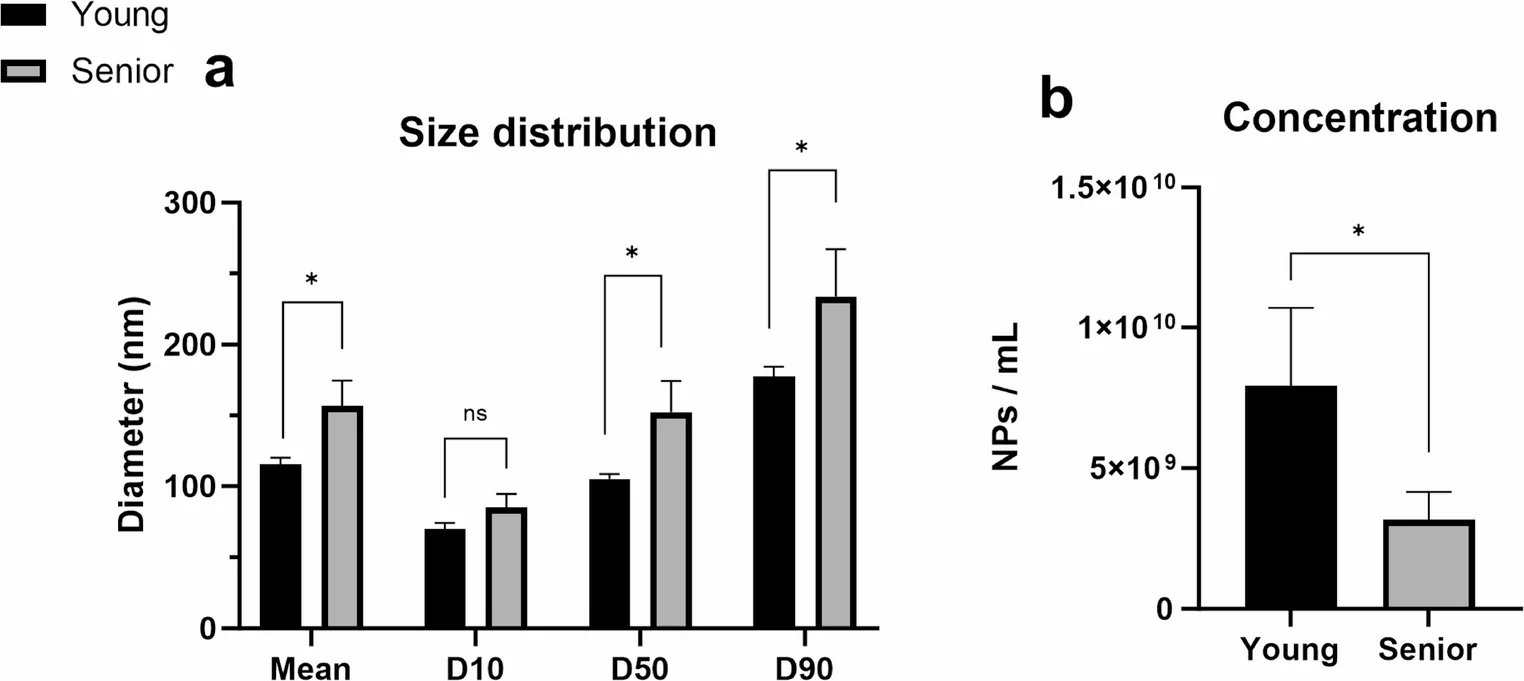
Senior dogs produce less and larger EVs than young dogs. Concatenated data from Fig. 1, showing average of nanoparticles (Np) from young and senior dogs’ plasma and the percentage of size ranges D10, D50, and D90 values, corresponding to the diameters below which 10%, 50%, and 90% of the particles are found, respectively (**a**). Total concentration of Nps from plasma young and senior dogs (**b**). Results are presented as the mean with standard error of the mean (SEM), and statistical analysis was conducted using an unpaired T-test; * indicates *p* < 0,05, and ns indicates non-significant difference

We also examined the average of EVs diameters and observed that the mean of EVs diameter in senior dogs was significantly higher than that in young ones. Particle size distribution was characterized by D10, D50, and D90 values, corresponding to the diameters below which 10%, 50%, and 90% of the particles are found, respectively, and we found that D50 and D90 were also higher in the senior group (Fig. 2a). At the same time, senior dogs presented significantly fewer EVs in plasma than young dogs (Fig. 2b).

### EVs from elderly dogs have more sterol than those from young dogs

**Fig. 3:**
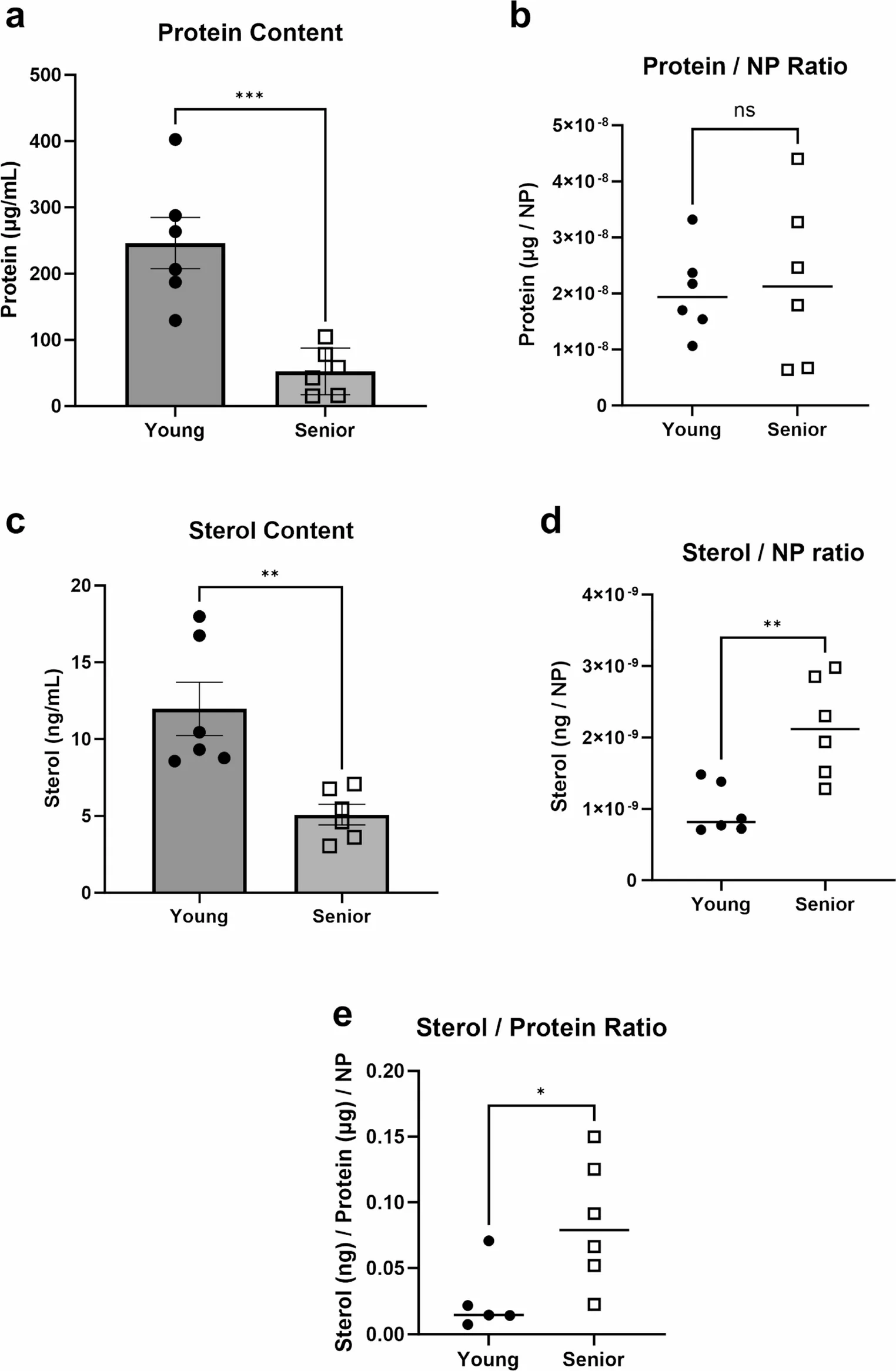
Senior dogs produce less EVs than young dogs. Protein (a and b) and sterol (c and d) content of isolated EVs from elder and young dogs’ plasma quantified using the commercial kits Micro BCA (protein) and Amplex Red Cholesterol (sterol). Panels **a** and **c** show the total concentration of protein and sterol in the same initial volume of plasma EVs. Panels **b**, **d** and **e** represent the normalized amount of protein and sterol in relation to the number of nanoparticles (Np) measured in the NTA equipment. Panel **e** shows the proportion between sterol and protein in each Np. Results are represented by individual values with standard error of the mean (SEM), and statistical analysis was conducted using an unpaired T-test; * indicates *p* < 0,05, ** indicates *p* < 0,005, and *** indicates *p* < 0,001, while ns indicates non-significant difference

Analyzing the total protein and sterol content of isolated EVs from the same initial plasma volume, we observed a significant reduction in the concentration of these molecules in the EVs from senior dogs compared to those from young dogs (Fig. 3a and c). This finding corroborates the NTA data (Figs. 1 and 2) and indicates that the total EVs concentration is reduced in elderly dogs.

Then, we analyzed the concentration of protein and sterol in relation to each individual sample of extracellular vesicle (EV) (Fig. 3b and d), normalizing the total amount of protein and sterol by dividing each value by the number of nanoparticles obtained from the NTA data. In this manner, we did not find any differences in the protein ratio (Fig. 3b); however, we observed a significantly higher concentration of sterol in EVs from elderly dogs compared to those from young dogs (Fig. 3d).

Additionally, we normalized sterol content relative to protein levels. The resulting sterol/protein ratio per EV was also higher in senior dogs (Fig. 3e). This finding may explain the higher prevalence of EVs with larger diameters in this group (Fig. 2), in which the sterol may be aggregating within the phospholipid membrane, prompting us to examine the EVs using microscopy.

**Fig. 4:**
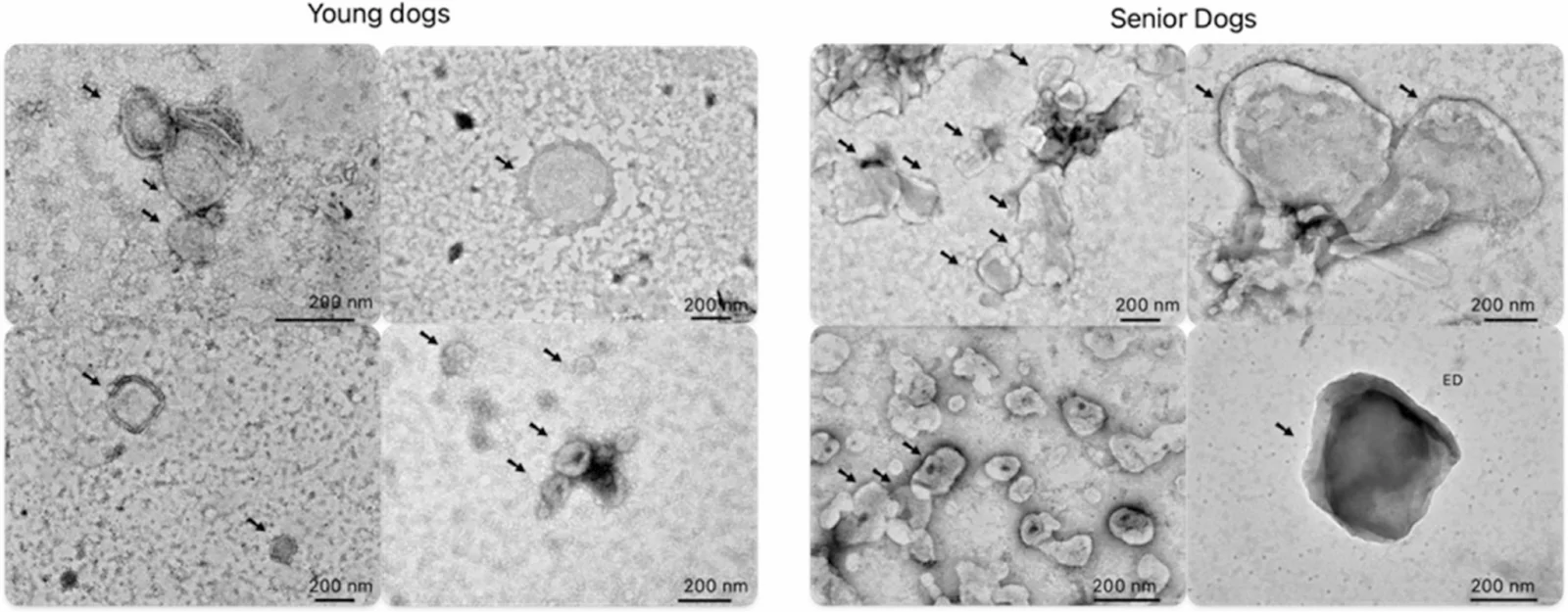
Representative TEM images of EV-enriched particles from young and senior dogs. Some EV-enriched particles from senior dogs appeared morphologically distinct in representative TEM images; however, quantitative ImageJ analysis did not detect significant differences in average size, membrane thickness, or electron density between groups

Finally, the microscopic analysis reinforced the previous results, showing a great diversity of EVs in terms of size, morphological characteristics, and electron density in the EVs from senior dogs, while those from younger ones were more homogeneous (Fig. 4), indicating differential content between groups (Gennebäck et al. 2013). We analyzed EV images using ImageJ; however, the results were inconclusive, with no significant differences observed between groups regarding average size, membrane thickness, or electron density. Nevertheless, it is important to emphasize that microscopy is primarily a qualitative rather than a quantitative method of analysis. Therefore, nanoparticle tracking analysis (NTA) and molecular content quantification are considered more reliable approaches for comparative evaluation.

### Plasma EVs from elderly dogs present a reduced content of miR-19b and miR-29c

**Fig. 5:**
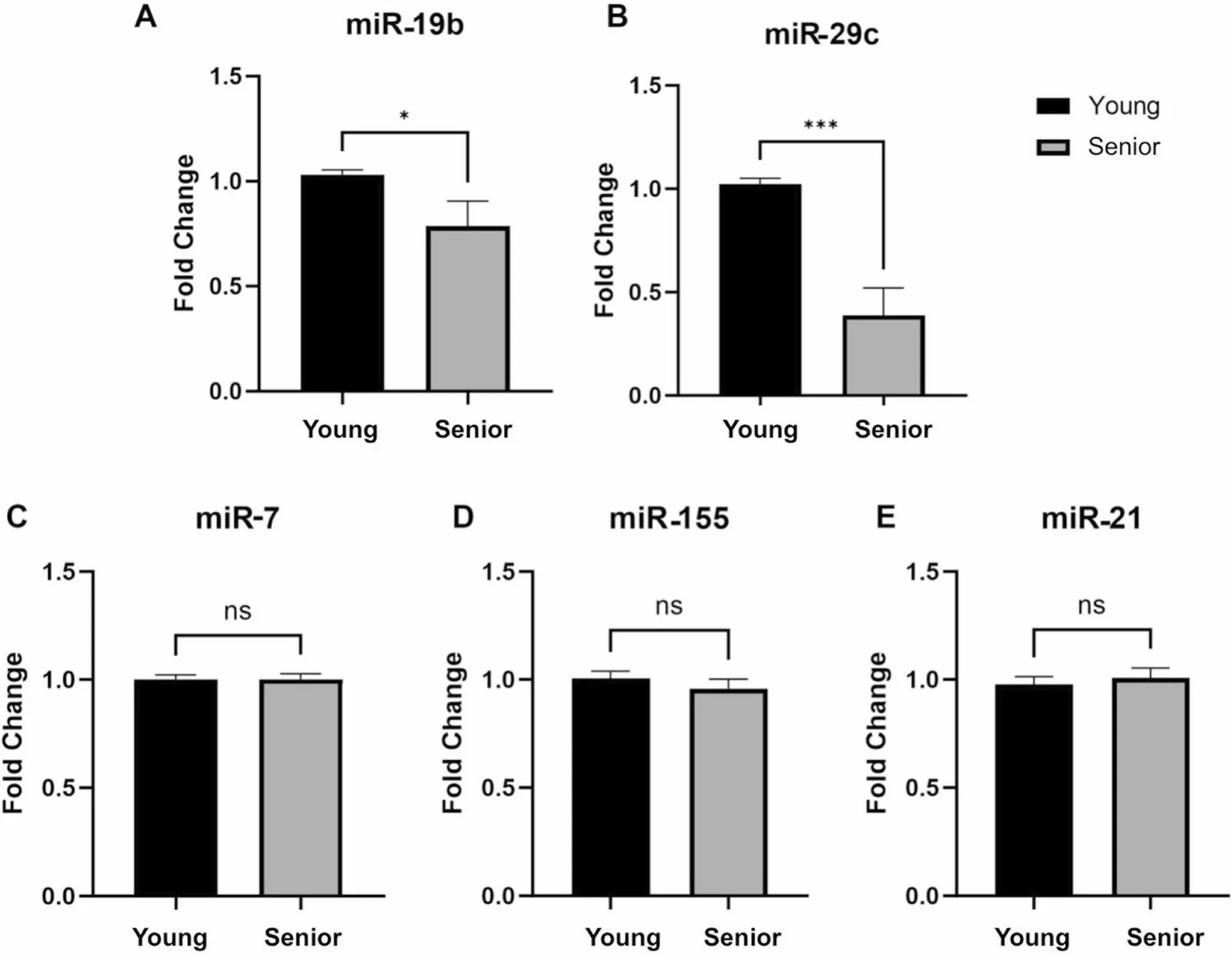
EVs from senior dogs contain less miR-19b and miR-29c than EVs from young dogs. Fold change of miRNAs miR-19b (A), miR-29c (B), miR-7 (C), miR-155 (D) e miR-21 (E) quantified by RT-qPCR isolated from EVs of young and senior dogs’ plasma. Results are represented by mean with standard error of the mean (SEM), and statistical analysis was conducted using an unpaired T-test; * indicates *p* < 0,05, *** indicates *p* < 0,001, and ns indicates non-significant difference

Considering the differential characteristics of dogs’ EVs by age, and the importance of these structures in cell-to-cell communication, we analyzed the content of regulatory RNAs, specifically microRNAs, which are typically enriched in exosomes. We selected five miRNAs previously related to neurodegenerative disorders. We observed that EVs from elderly dogs contained significantly lower levels of miR-19b and miR-29c than those from younger dogs. The fold change values were 0.8 and 0.38 for miR-19b and miR-29c, respectively. In contrast, miR-7, miR-155, and miR-21 were not differentially expressed between the groups (Fig. 5).

Taking together, our results reinforce the importance of EVs in transporting diverse molecules throughout the organism, including miRNAs. They also highlight that their morphological characteristics and internal content may influence the aging process.

## Discussion

Comparing the characteristics of EVs from young and senior dogs is important for highlighting aging biomarkers in this species for identifying potential targets for the development of more effective treatments to enhance the quality of life for elderly pets. Studies on senescence in animals can also contribute to understanding this process in humans, given the population’s increased life expectancy. Indeed, EVs are notably significant cellular components due to their contrasting roles in health and disease. EVs exhibit substantial plasticity in their functions, mediating the molecular secretome, intercellular communication, and signal transmission. Conversely, EVs may play a crucial role in dysregulation of the aging machinery (Théry et al. 2018, Hamdan et al. 2021).

Our study demonstrated that elderly dogs contain fewer serum EVs than young dogs. This finding is consistent with previous studies involving human plasma, which reported a reduction in exosome concentration with age progression (Eitan et al. 2017). Eitan et al. suggested that this reduction may be attributed to an increase in the internalization of circulating exosomes by B cells, rather than a decrease in production by the cells of older individuals (Eitan et al. 2017). In fact, it has been shown that epithelial senescent cells release more exosomes than their young counterparts, accompanied by an increased expression of exosome markers RAB7 and CD63, as well as elevated lysosome content in their cytoplasm (Riquelme et al. 2020). The increase in surface markers may explain the elevated exosome internalization by B cells, followed by increased MHCII expression on monocytes. Interestingly, pre-activation with LPS improved the uptake of exosomes by B cells, mimicking an inflammatory condition commonly observed in the elderly (Riquelme et al. 2020).

The inflammatory microenvironment is initiated by the senescence-associated secretory phenotype (SASP), in which senescent cells secrete a range of pro-inflammatory molecules, including cytokines, chemokines, matrix proteases, and growth factors. These molecules, mainly transported by exosomes, promote the proliferation of neighboring cells and induce tumorigenic effects in pre-malignant recipient cells (Takasugi et al. 2017, Wang et al. 2024). In contrast, it has been demonstrated that mesenchymal stem cells secrete the rejuvenating factor GDF-11 within exosomes, promoting the polarization switch of macrophages from M1 to M2, thereby enhancing tissue regenerative capacity (Idkowiak-Baldys et al. 2019).

Our study demonstrated that EVs from senior dogs exhibit greater polydispersity in size, with EVs from senior dogs averaging larger than those from young dogs. The increased size and elevated sterol content in EVs from senior dogs indicate a higher concentration of LDL in these structures (Sódar et al. 2016). Previous study comparing exosomes from endothelial senescent cells and those from young cells did not find differences in size or morphological characteristics (Riquelme et al. 2020). Nonetheless, Eitan et al. reported differential protein content in plasma exosomes from older and young donors, as measured by ELISA. In exosomes from older individuals, they observed reduced levels in apoptosis markers, including p53, cleaved PARP, and Caspase-3, along with increased levels of proteins related to tumorigenesis and metastasis, specifically CD151 and MUCIN16 (Eitan et al. 2017). The absence of specific exosomes markers in our study, such as CD63, CD9, CD81, prevent us to confidently affirm that the analyzed structures are purified exosomes, but EV-enriched preparations. However, EVs are an heterogeneous group of bilayer-enclosed particles including exosomes, micro-vesicles, liposomes, with different roles in the organism and we acknowledge that our data represent all those structures (Kumar et al. 2024).

Considering the importance of EVs for cellular communication, the transport of miRNA is essential for regulating gene expression and maintaining homeostasis, thereby enabling a refined global protein synthesis (Eiring et al. 2010). Multiple groups have demonstrated that miRNAs modulate biological and, unfortunately, pathological aging, including by directly influencing telomere shortening and ROS production (Zhang et al. 2019, Bai et al. 2011, Lang et al. 2016, Hafner et al. 2010, Hrdličková et al. 2014, Slattery et al. 2016, Vinchure et al. 2021, Li et al. 2020). However, the regulation of these functions through cell-to-cell signaling relies on the transport of microRNAs within EVs, which promotes their efficient delivery to distant organs and tissues.

Previous studies have shown that specific miRNAs, such as miR-21, miR-29, and miR-34, are involved in tissue regeneration and homeostasis, potentially impacting life expectancy (Hamdan et al. 2021). miR-29c and miR-19b, which were down-regulated in EVs from senior dogs in the present work, were previously reported to be down-regulated in patients with Parkinson’s disease and other neurodegenerative disorders that exemplify pathological aging (Gui et al. 2015, Bai et al. 2021). Controversially, earlier studies showed that free miR-29 increases in multiple tissues with aging, and its overexpression induces accelerated aging-related phenotypes (Swahari et al. 2024, Takeda and Tanabe 2016). This discrepancy suggests that more miR-29 is delivered to tissues in the elderly, while it is reduced in blood EVs. These data indicate that miRNA expression varies across tissues and life phases. For instance, miR-19 level increases in plasma from individuals around 70 years old but decreases in plasma from centenarians (Morsiani et al. 2021). Additionally, the expression of miR-19 decreases with age in bone samples from mice and in posterior iliac crest bone biopsies from healthy female donors. This miRNA suppresses the expression of p16^Ink4a^ and p21^Cip1^, thereby influencing the rise of senescence phenotypes (Kaur et al. 2023).

Finally, our study demonstrated significant differences in the content and concentration of plasma EVs from young and senior dogs. These findings suggest that plasma EVs represent promising biomarkers of aging, given the ease of sample collection and processing and the extensive information they provide on organ homeostasis and associated gene expression patterns. This information is crucial for assessing the organism’s overall health and identifying biomarkers indicative of disease and pathological aging before symptom intensification. Although this study presents a promising strategy for diagnosing pathological aging, the small sample size limits the generalizability of our findings. A limitation of the study is the difficulty of attract dogs donors, specially the elder ones, which are less common, more fragile and with limited vein exposure for blood collection, what make the owners more worried with their dogs, explaining the small experimental number. The animals evaluated may not represent the broader dog population, highlighting the need for studies with larger sample sizes that include diverse geographic regions and a group with intermediate age, also focusing on specific variations among different breeds.

## Data Availability

The datasets generated during and analysed during the current study are not publicly available due to ongoing analyses and related unpublished work, but are available from the corresponding author on reasonable request.
